# Natural Antispasmodics: Source, Stereochemical Configuration, and Biological Activity

**DOI:** 10.1155/2018/3819714

**Published:** 2018-10-08

**Authors:** Edith Fabiola Martínez-Pérez, Zaida N. Juárez, Luis R. Hernández, Horacio Bach

**Affiliations:** ^1^Department of Medicine, Division of Infectious Diseases, University of British Columbia, 2660 Oak Street, Vancouver, BC, Canada V6H 3Z6; ^2^Departamento de Ciencias Químico-Biológicas, Universidad de las Américas Puebla, Ex Hacienda Sta. Catarina Mártir S/N, 72810 San Andrés Cholula, PUE, Mexico; ^3^Ingeniería en Biotecnología, Facultad de Biotecnología, Decanato de Ciencias Biológicas, Universidad Popular Autónoma del Estado de Puebla, 21 Sur No. 1103, Barrio Santiago, 72410 Puebla, PUE, Mexico

## Abstract

Natural products with antispasmodic activity have been used in traditional medicine to alleviate different illnesses since the remote past. We searched the literature and compiled the antispasmodic activity of 248 natural compounds isolated from terrestrial plants. In this review, we summarized all the natural products reported with antispasmodic activity until the end of 2017. We also provided chemical information about their extraction as well as the model used to test their activities. Results showed that members of the Lamiaceae and Asteraceae families had the highest number of isolated compounds with antispasmodic activity. Moreover, monoterpenoids, flavonoids, triterpenes, and alkaloids were the chemical groups with the highest number of antispasmodic compounds. Lastly, a structural comparison of natural versus synthetic compounds was discussed.

## 1. Introduction

Antispasmodic compounds are currently used to reduce anxiety, emotional and musculoskeletal tension, and irritability. Although most of the available antispasmodic compounds are synthetic or semisynthetic, traditional uses of this group of compounds are still popular.

We collected information about natural compounds with antispasmodic activity isolated from terrestrial plants. We searched the databases of Google Scholar, PubMed, and SciFinder and compiled the information about 248 compounds published until December 2017. This review focuses on the antispasmodic activity of isolated compounds and activities from extracts without further purification are not discussed.

## 2. The Neurons

Nerve cells or neurons are responsible for receiving, conducting, and transmitting signals. A neuron consists of a nucleated body, a long thin extension called an axon, and several dendrites or prolongations extended from the cell body. Axons conduct signals from the nucleated body towards distant targets, while dendrites provide an enlarged surface area to receive signals from the axons of other neurons.

Signal transmission through axons is driven by a change in the electrical potential across the plasma membrane of neurons. This plasma membrane contains voltage-gated cation channels, which are responsible for generation of action potentials. An action potential is triggered by a depolarization of the plasma membrane or a shift to a less negative value.

In nerve and skeletal muscle cells, a stimulus can cause sufficient depolarization to open voltage-gated Na^+^ channels allowing the entrance of Na^+^ into the cell. This influx of Na^+^ depolarizes the membrane further causing the opening of more Na^+^ channels. To avoid a permanent influx, Na^+^ channels are able to reclose rapidly even when the membrane is still depolarized. This function is based on the presence of voltage-gated K^+^ channels, which are responsible for K^+^ efflux equilibrating the membrane potential even before the total inactivation of Na^+^ channels. In some cases, the action potential in some muscles depends on voltage-gated Ca^2+^ channels.

### 2.1. Transmission of Signals

The transmission of signals occurs mainly between neurons or from neurons to skeletal muscles, which are the final acceptors of electrical signals, causing a muscular contraction.

#### 2.1.1. Signal Transmission between Neurons

Neuronal signals are transmitted between neurons at specialized sites of contact known as synapses. Neurons are separated by a synaptic cleft where a release of a neurotransmitter occurs. This neurotransmitter is stored in vesicles and is released by exocytosis. Upon triggering, the neurotransmitter is released into the cleft provoking an electrical change in the postsynaptic cell by binding to the transmitter-gated ion channels. To avoid a continuous electrical change and to ensure both spatial and temporal precision of signal transmission, the neurotransmitter is rapidly removed from the cleft either by specific enzymes in the synaptic cleft or by reuptake mediated by neurotransmitter carrier proteins [1].

Neurotransmitters can also open cation channels causing an influx of Na^+^ and then called excitatory neurotransmitters (e.g., acetylcholine, glutamate, and serotonin) or produce an opening of Cl^−^ channels and then inhibiting the signal transmission by maintaining the postsynaptic membrane polarization [e.g., *γ*-aminobutyric acid (GABA) and glycine].

#### 2.1.2. Neuromuscular Signal Transmission

The transmission of electrical signals to muscles involves five sequential and orchestrated steps: (i) nerve electric signal reaches the nerve terminal, (ii) it depolarizes the plasma membrane of the terminal, (iii) voltage-gated Ca^2+^ channels opens causing an increase in Ca^2+^ concentration in the neuron cytosol, and (iv) release of acetylcholine into the synaptic cleft is triggered. Acetylcholine binds to acetylcholine receptors in the muscle plasma membrane opening Na^+^ channels and provoking a membrane depolarization. This depolarization enhances the opening of more Na^+^ channels causing a self-propagating depolarization. The generalized depolarization of the muscle plasma membrane activates Ca^2+^ channels in specialized regions on the membrane causing Ca^2+^ release from the sarcoplasmic reticulum (Ca^2+^ storage) into the cytosol.

As a consequence of an increase in the Ca^2+^ concentration, myofibrils in the muscle cell contract. The increase of Ca^2+^ in the cytosol is transient because Ca^2+^ is rapidly pumped back into the sarcoplasmic reticulum causing a relaxation of the myofibrils. This process is very fast and Ca^2+^ concentration at resting levels is restored within 30 milliseconds [2].

## 3. Receptors

The autonomic nerve system controls and monitors the internal environment of the body. The input of its activity is provided by neurons that are associated with specific sensory receptors located in the blood vessels, muscles, and visceral organs (Table 1). According to the neurotransmitter secreted, these neurons are classified as adrenergic or cholinergic. The adrenergic neurons secrete the neurotransmitter noradrenalin termed also norepinephrine. Adrenergic receptors include the types *α* and *β*, which are further categorized as *α*_1_, *α*_2_, *β*_1_, *β*_2_, and *β*_3_. On the other hand, cholinergic neurons secrete acetylcholine, which induces a postsynaptic event. There are two types of cholinergic receptors, the nicotinic receptor (abundant at the neuromuscular junction) and the muscarinic receptor (abundant on smooth and cardiac muscles and glands).

There are several agonists (neurotransmitters, hormones, and others) able to bind to specific receptors and activate the contraction of smooth muscle. Upon binding the agonist to the receptor, the mechanism of contraction is based on an increase of phospholipase C. This enzyme hydrolyzes phosphatidylinositol 4,5-bisphosphate located on the membrane, producing two powerful secondary messengers termed diacylglycerol (DG) and inositol 1,4,5 triphosphate (IP3). IP3 binds to specific receptors in the sarcoplasmic reticulum, causing release of Ca^2+^ within the muscle. DG together with Ca^2+^ activates the protein kinase C (PKC), which phosphorylates specific proteins. In most smooth muscles, the contraction process commences when PKC phosphorylates Ca^2+^ channels or other proteins that regulate the cyclic process. For instance, Ca^2+^ binds to calmodulin (a multifunctional intermediate calcium-binding messenger protein), triggering the activation of the myosin light chain (MLC) kinase, which phosphorylates the light chain of myosin and together with actin carries out the process of initiating the shortening of the smooth muscle cell [147]. However, the elevation of the intracellular concentration of Ca^2+^ is transient, and the contractile response is maintained by a mechanism sensitized by Ca^2+^ modulated by the inhibition of myosin phosphatase activity by Rho kinase. This mechanism sensitized to Ca^2+^ is initiated at the same time that phospholipase C is activated and involves the activation of the small RhoA protein bound to guanosine triphosphate (GTP). Above activation, RhoA increases the activity of Rho kinase, leading to the inhibition of myosin phosphatase. This promotes the contractile state, since the myosin light chain cannot be dephosphorylated [147].

Relaxation of smooth muscle occurs as a result of either removing the contractile stimuli or by the direct action of a substance that stimulates the inhibition of the contractile mechanism. In any circumstance, the relaxation process requires a decrease in the intracellular Ca^2+^ concentration and an increase in the activity of the MLC phosphatase. The sarcoplasmic reticulum and plasma membrane remove Ca^2+^ from the cytosol. Na^+^/Ca^2+^ channels are located on the plasma membrane and help to reduce the intracellular concentration of Ca^2+^. During relaxation, other contributors that restrict the Ca^2+^ entry into the cell are the voltage-operated channels and Ca^2+^ receptors in the plasma membrane, which remain closed [147].

## 4. Spasmodic Compounds

The historical antecedents date from the year 1504 when South American natives inhabiting the basins of the high Amazon and the Orinoco prepared a mixture of alkaloids termed curare. This substance was placed in the tips of arrows in order to hunt (prey paralyzing) and fight in wars. Curare produces muscle weakness, paralysis, respiratory failure, and death [148]. In 1800, Alexander von Humboldt, identified that curare was made from the extracts of the species* Chondrodendron tomentosum* and* Strychnos toxifera*.

In 1935, the French physiologist Claude Bernard managed to isolate the alkaloid d-tubocurarine from the curare [149]; and one year later, it was elucidated that this compound had the ability to inhibit acetylcholine, blocking the transmission of nerve impulses to the muscles [150]. Lastly, new benzylisoquinoline alkaloids were isolated from curare by Galeffi et al. in 1977 [151, 152].

In 1822, the pharmacist Rudolph Brandes obtained an impure alkaloid from* Atropa belladonna* (Solanaceae), which after purification was named atropine. Interestingly, atropine was not produced as a natural compound from the plant and it was a derivative generated from the alkaloid hyoscyamine during the process of purification [153]. It is important to note that atropine has been naturally found in small quantities in other members of the Solanaceae family such as* Datura stramonium*,* Duboisia myoporoides*, and* Scopolia japonica* [154–156].

The use of the plant* Papaver somniferum* (opium poppy) (Papaveraceae) dates back to about 4000 BC. At present the plant is only used to extract a base material for the manufacture of other alkaloids, such as noscapine and codeine, both discovered by the French pharmacist Pierre-Jean Robiquet in 1831 and 1832, respectively [157]. In 1848, papaverine was another substance extracted from the same plant by the German chemist Georg Merck [158], which is rarely used today because of the high doses needed (approximately 6 to 12 mg). However, it is still used as a control in experimental models with the purpose of studying antispasmodic activity of plant extracts.

In the 20^th^ century, extracts and powders derived from* A. belladonna* were widely used as antispasmodics, but from the 1950s these preparations were displaced by synthetic and semisynthetic anticholinergic compounds in order to obtain a better response [159], such as the case of methocarbamol and guaifenesin. On the other hand, a series of compounds such as dantrolene, glutethimide, methaqualone, chlormezanone, metiprilone, and ethchlorvynol were introduced to replace the meprobamate, which had to be withdrawn from the market in 1960 due to problems resulting from use such as abstinence, addictions, and overdoses.

In 1962, the Swiss chemist Heinrich Keberle synthesized baclofen, which can be obtained by reacting glutarimide with an alkaline solution [160]. Glutarimide can also be found in plants such as* Croton cuneatus* and* C. membranaceus* (Euphorbiaceae) [161, 162].

The arrival of the quaternary compounds of nitrogen reinforce their peripheral anticholinergic activity offering also the advantages of being poorly absorbed in the gastrointestinal tract, producing a more powerful and longer lasting sedative effect unlike atropine [1]. For example, ipratropium bromide was developed by the German company Boehringer Ingelheim in 1976 and used to treat asthma. This compound was obtained by reacting atropine with isopropyl bromide [163]. Another quaternary compound was the n-butylhyoscine bromide, which is possible to obtain by the organic synthesis of scopolamine and the cimetropium bromide found in the* A. belladonna* [164]. Although at present the preparations of plant mixtures are no longer used for therapeutic purposes, these compounds formed a part of and served as the basis for modern pharmacology for their applicability as antispasmodics and anesthetics.

Spasms are involuntary contractions of the muscles, which are normally accompanied by pain and interfere with the free and effective muscular voluntary activity. Muscle spasm can originate from multiple medical conditions and is often associated with spinal injury, multiple sclerosis, and stroke.

Spasticity and rigidity are caused by a disinhibition of spinal motor mechanisms. There are several scenarios where a muscle can produce a spasm: (i) unstable depolarization of motor axons; (ii) muscular contractions persist even if the innervation of muscle is normal and despite attempts of relaxation (myotonia); (iii) after one or a series of contractions, the muscle can decontract slowly, as occurring in hypothyroidism; and (iv) muscles lack the energy to relax.

### 4.1. Distribution of Spasmodic Compound in Nature

Spasmodic compounds are widely distributed in nature (Table 2). Frequently, these compounds are found in animals that paralyze their preys or used for defense. Some examples include the venom of the black widow and tarantula spiders [11, 165] and the venom of snakes [166]. Plants also produce spasmodic metabolites, such as strychnine, an alkaloid obtained from the tree* Strychnos nux-vomica* (Loganiaceae). Furthermore, microorganisms synthesize spasmodic compounds such as the neurotoxins tetanospasmin and botulinum toxin from the Gram-positive bacteria* Clostridium tetani *and* C. botulinum*, respectively. These toxins produce a toxic disorder, which is characterized by persistent spasms of skeletal muscles on spinal neurons similar to strychnine.

### 4.2. Mechanisms of Antispasmodic Activity of Natural Products

Antispasmodic compounds exert their activity in different ways, such as antispasmodic activity through inhibition of the response to the neurotransmitters 5-hydroxytryptamine (5-HT) or serotonin and acetylcholine. However, other authors attribute the antispasmodic effect to (i) capsaicin-sensitive neurons, (ii) the participation of vanilloid receptors [167], (iii) the activation of K^+^ ATP channels, (iv) the blockade of Na^+^ channels and muscarinic receptors, (v) the reduction of extracellular Ca^2+^, or (vi) the blockade of Ca^2+^ channels [22, 168, 169]. The above is merely a reflection of the ambiguity of the studies showing the mechanisms of action of the antispasmodic compounds [36]. For example, the hydroalcoholic extract of* Marrubium vulgare* showed antispasmodic effect, having the ability to inhibit the neurotransmitters acetylcholine, bradykinin, prostaglandin E2, histamine, and oxytocin [170], whereas a dual effect of antidiarrheal and laxative activities was reported in* Fumaria parviflora* [171].

## 5. Methods Used to Evaluate Antispasmodic Compounds

### 5.1. Gastrointestinal Model

The small intestine is characterized by its large surface area as a result of its circular folds, villi, and microvilli. It is the longest part of the GI system (approximately 5 meters) and comprises about 5% of its initial length, which corresponds to the duodenum (characterized by the absence of the mesentery) and then the jejunum (around 40% of the intestinal length), ending with the ileum. It is the organ of absorption of nutrients and digestion in organisms. These functions are carried out mainly in the duodenum and jejunum.

The main types of bowel movement are the segmentation and peristaltism. The segmentation is most frequent in the small intestine and consists of contractions of the circular muscle layer in very close areas. Contractions last for 11-12 and 8-9 contractions per min in the duodenum and ileum, respectively. When this segmentation is rhythmic, the contractions are alternated with relaxation. This type of movement results in a mixed effect of the chyme (acidic fluid that passes from the stomach to the small intestine) with the digestive secretions, allowing an optimal contact with the intestinal mucosa. In the case of peristalsis, contractions of successive sections of the circular smooth muscle cause the movement of the intestinal contents in anterograde form. The short peristaltic movement also takes place in the small intestine, but less frequently than the segmentation movements. Peristaltic waves rarely cross more than 10 cm of intestine and, due to the low frequency of propulsion of the chyme, it is in this zone where digestion and absorption are preferably carried out. Peristalsis is regulated mainly by the nervous action of the myenteric plexus (major nerve supply to the gastrointestinal tract that controls GI tract motility) in the intestinal wall.

The diversity of experimental models used for the testing of antispasmodic compounds is large. These models mainly use isolated organs or live animals. Once the organ is extracted from the animal, the intestinal motility is assessed with the administration of a substance. The use of extracted organs can be sustained for hours when placed in a physiological solution, such as Ringer, Jalon, Tyrode, and Krebs [172].

The most used organs to perform the studies are guinea pig ileum, duodenum, heart, trachea, and jejunum. The same organs can be also extracted from rabbit, mouse, rat, and hamster (Table 3). The preparation of ileum is preferred because it evaluates the spasmolytic activity. However, although the jejunum contracts spontaneously, it allows evaluating the spasmolytic activity directly and without the use of an agonist [173].

Some advantages of performing* ex vivo* experiments are as follows: (i) different substances can be evaluated in fresh tissues without absorption factors, metabolic excretion or interference due to nerve reflexes; (ii) it is possible to quantify the effect produced by a precisely determined drug; and (iii) it is easier to obtain dose-effect curves, such as the smooth muscle where the contraction obtained under the influence of a spasm or in tissue homogenates is measured by determination of the enzyme activities [172, 174].

### 5.2. Guinea Pig Ileum and Rat Stomach

The ileum is removed and cut in strips of approximately 2 cm long and then placed in a bath filled with an isotonic solution as mentioned earlier. Electrophysiological studies are performed by graphically recording the contractions with the aid of a transducer, which is calibrated 30 min before the treatment begins. A range of 0.01 to 0.03 *μ*M is generally used to determine dose response curves of the antispasmodic substance [175].

In rats, the stomach is removed and the corpus and fundus are cut in strips of approximately 5 mm x 15 mm and placed on a prewarmed warm solution as mentioned before.

### 5.3. Compounds Used to Elicit a Spasmodic Activity

The main compounds used are acetylcholine, atropine, BaCl_2_, carbachol, histamine, KCl, and serotonin.

Acetylcholine is a postganglionic neurotransmitter in the parasympathetic neurons that innervate the intestine. The response to acetylcholine is regulated by activation of the two types of muscarinic receptors: M2 and M3 [176]. The activation of these receptors causes contractions by increasing the intracellular concentration of Ca^2+^ via IP3 [176]. Atropine is a competitive reversible antagonist of muscarinic acetylcholine receptors M1, M2, M3, M4, and M5.

Different substances are used to produce contractions. For example, BaCl_2_ induces contractions by mobilizing membrane-bound Ca^2+^ [177], carbachol is a cholinomimetic drug (cholinergic agonist) that binds and activates acetylcholine receptors [178], histamine acts by either accelerating the release of acetylcholine or interacting supra-additively with the acetylcholine at the smooth muscle [179], whereas KCl increases the voltage-operated Ca^2+^ channel activity by increasing intracellular free Ca^2+^ in smooth muscle [180]. Serotonin is also an important neurotransmitter mainly stored in the digestive tract, affecting the secretory and motor activities. At high concentrations, it acts as a vasoconstrictor by contracting endothelial smooth muscle directly or by potentiating the effects of other vasoconstrictors [181, 182].

## 6. Antispasmodic Activity of Natural Compounds

**Figure 1:**
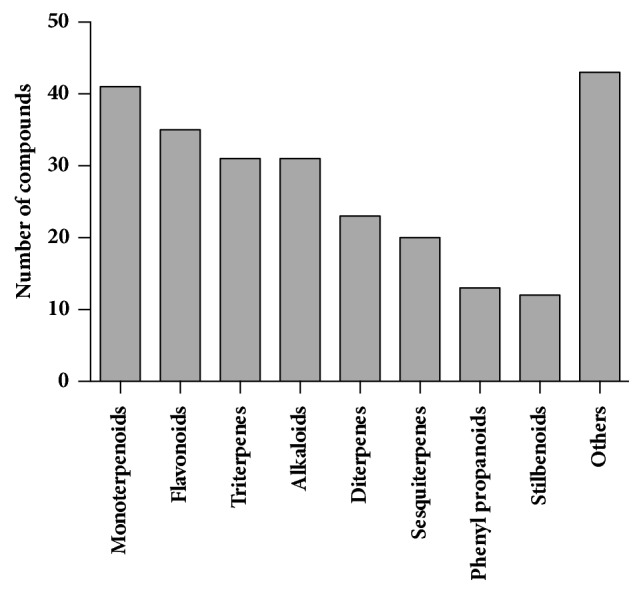
**Number of isolated compounds with antispasmodic activity.** The total number was obtained from Table 3. “Others” is the sum of the compounds belonging to alcohols, amines, benzofurans, chalcones, coumarins, curcuminoids, isothiocyanates, ketones, phenolic, phenylmethanoids, phenylethanoids, glucinols, and phloroglucinols.

Compounds isolated from terrestrial plants have shown the ability to function as antispasmodic compounds. The chemical group with the highest number of members of antispasmodic compounds is the monoterpenoid group (41 compounds) followed by flavonoids (35 compounds), alkaloids (with 33 compounds), and triterpenes with 31 (Figure 1). Although we summarize in Table 3 248 compounds, in most of the cases the mechanism behind their activity has not been elucidated.

## 7. Mutagenicity

Studies related to the mutagenicity of antispasmodics are very scarce. This topic has been underestimated when testing the bioactivities of ethnomedicinal plants. Probably the most useful method to determine the mutagenicity of natural products or plant extracts is the Ames method [183]. This test is based on the rate of mutations detected in genetically modified strains of* Salmonella typhimurium. *Moreover, this test has also been developed to detect mutagenicity of metabolized compounds in the liver. In this situation, a mixture of liver enzymes (S9 microsomal fraction) is used to mimic the metabolites that will be produced in the liver [184].

Few studies have been performed to determine the mutagenicity of natural products with antispasmodic activity. For example, the flavonoids quercetin and luteolin were tested using the Ames method and the appearance of point mutations in four of the tested bacterial strains was shown [185]. In another study, the extracts of the plants* Brickellia veronicaefolia*,* Gnaphalium* sp.,* Poliomintha longiflora*, and* Valeriana procera* were studied. Compounds isolated from these plants are listed as antispasmodic compounds (Table 3). Results of the mutagenicity test indicated that* Gnaphalium* sp.,* Poliomintha longiflora* (used in the Mexican cuisine and as a traditional medicine), and* Valeriana procera* induced mutagenesis in the tested bacterial strain [186].

## 8. Chemical Similarities between Natural and Synthetic Antispasmodic Compounds

To determine whether or not there is an analogy between synthetic (Table 4) and natural antispasmodic compounds, the structures of both groups were compared. Results showed that no similarities were found except for alkaloids, amines, and amino acids.

One of the main differences is that commercial alkaloids are methylated in their nitrogen to make them positive, increasing their solubilities because of salt formation. In contrast, natural products have no positive nitrogen, rendering the molecule neutral and pH dependent. Thus, the compound may or may not be protonated, resulting in a change in its solubility and consequently a change on the targeting tissues.

The comparison can perhaps be focused on the distribution of charges rather than by functional groups or families of compounds, emphasizing the electron distribution. For example, a physical characterization such as the heat of formation, the surface electrostatic potential, the molecular weight, the surface tension, the refractive index, the lipophilicity, and others has been used to characterize the structure-activity relationship of alkaloids extracted from the Amaryllidaceae family [187]. These alkaloids were selected because of their ability to inhibit the effect of the acetylcholinesterase enzyme.

Of special interest is the natural compound salvinorin A isolated from the Mexican hallucinogenic* Salvia divinorum *(Lamiaceae) used in the traditional medicine as an antidiarrheal. It has been reported that this compound inhibited the intestinal motility through the activation of other receptors such as *κ*-opioid receptors (KORs). Upon inflammation of the gut, the cannabinoid C, B_1_, and KOR receptors are upregulated. It appears that salvinorin A interacts in the cross-talk between these receptors with a reduction of the inflammation as demonstrated in murine and guinea pig models [188, 189].

Analysis of the similarities between synthetic and natural antispasmodic structures is depicted in Table 5.

## 9. Conclusions

A large number of natural products with antispasmodic activities have been reported. Although the use of plants in traditional medicine is still relevant, it is necessary to perform new studies to elucidate the mechanism of action of antispasmodics. Moreover, more information about cytotoxicity and mutagenesis should be explored to ensure that these compounds are safe for consumption. The findings of this study corroborated the need for safety studies on plants extensively used for primary health care in countries such as Mexico. Such studies must be carried out before continuing with the widespread use of some species, which may provoke long-term and irreversible damage.

## Figures and Tables

**Table 1:** Receptors targeted by neurotransmitters in the body.

Receptor	Targeted by	
Adrenergic	Epinephrine (adrenaline)	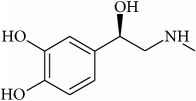
	Norepinephrine (noradrenaline)	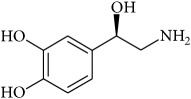
Dopaminergic	Dopamine	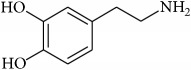
Cholinergic	Acetylcholine	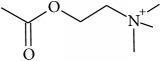
GABAergic	GABA	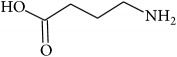
Glutaminergic	Glutamate	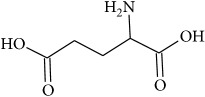
Histaminergic	Histamine	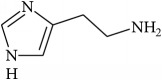
Serotonergic	Serotonin	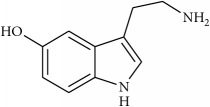
Glycinergic	Glycine	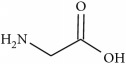
Opioid	Dynorphin	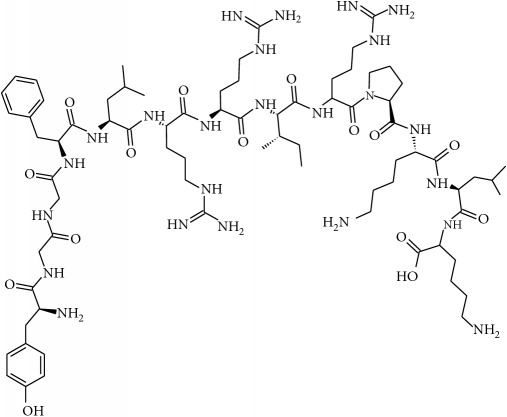
	Enkephalin	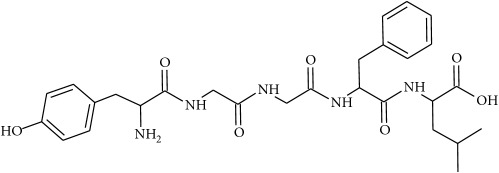
	Endorphin	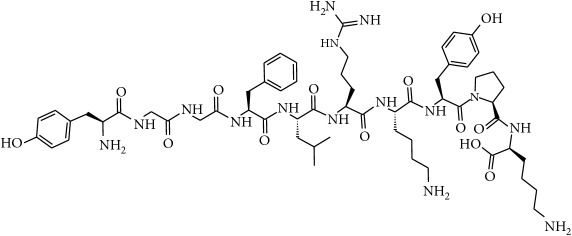
	Endomorphin	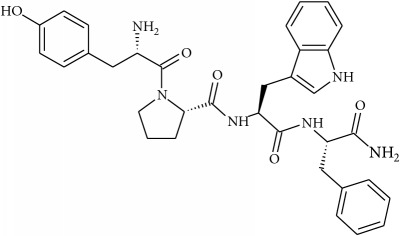
	Nociceptin	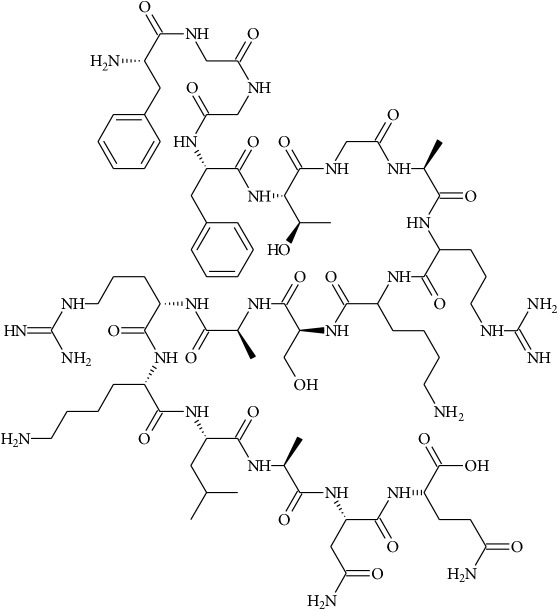

**Table 2:** Representative organisms producing spasmodic compounds.

**Compound**	**Organism**	**Symptoms**	**Mechanism**	**Reference**
***Bacterial***				
Botulinum toxin	*Clostridium botulinum*	Muscular relaxation	Secretion of acetylcholine into synapses is blocked	[3]
Tetanospasmin	*Clostridium tetani*	Muscular spasm	Inhibits the binding of GABA and glycine	[4]

***Marine***				
Nematocyst venom extract	Sea anemones	Nausea, vomiting, muscle cramp, severe pain, paralysis	Delay in the voltage-dependent Na^+^ channels inactivation	[5]
Nematocyst venom extract	*Chironex fleckeri* (Cnidaria)	Contraction of arterial smooth muscle	Increase of cytosolic Ca^2+^ concentration	[6]
Ciguatoxin	*Gambierdiscus toxicus* (Dinoflagellate)	Nausea, vomiting, abdominal pain, intestinal spasm	Interact with voltage-gated increasing the Na^+^ permeability and Ca^2+^ homeostasis	[7]
Chordata	*Plotosus lineatus* (Catfish)	Violent pain, shock, spasm	Increase of the vascular permeability in peritoneum	[8]

***Terrestrial***				
Ergotamine	*Claviceps purpurea *(fungus)	Seizure, spasms psychosis, nausea, vomiting	Agonist of several neurotransmitter receptors	[9]
*α*-Latrotoxin	*Latrodectus tredecimguttatus* (black widow spider)	Facial flushing, hypertension, muscle spasm, tachycardia	Causes Ca^2+^-dependent and -independent release of neurotransmitters	[10]
Vanillo-toxin, hanatoxin, huwentoxin	Tarantula species	Severe pain, cramps, erythema, swelling, tachycardia	Unrevealed	[11–14]
*β*-Neurotoxin	*Mesobuthus martensii *(scorpion)	Increases muscular contraction, spasm, convulsion	Modulates Ca^2+^ channels	[15]
Crotoxin	*Crotalus durissus terrificus *(rattlesnake)	Severe pain, drooping eyelids, low blood pressure, muscle weakness	Blocks the cholinergic post-synaptic response	[16]

**Table 3:** Natural products with antispasmodic activity isolated from terrestrial plants.

**Compound name**	**Species (Family)**	**Preparation (Solvent)**	**Model tested**	**Source**	**Reference**
*Monoterpenoids*					
**1** Myrcene, *β*-myrcene	*Plectranthus barbatus* (Lamiaceae)	Leaf (MeOH)	ACh, BaCl_2_, KCl in guinea pig ileum	EO	[17]
**2** Citral B, *β*-citral, Neral	*Aloysia triphylla* (Verbenaceae)	Leaf (Hexane)	Carbachol, KCl, O, PGF (2*α*) in rat uterus	IC	[18]
	*Cymbopogon citratus* (Poaceae)	Leaf (MeOH 70%)	ACh, KCl in rabbit ileum	IC	[19]
	*Melissa officinalis* (Lamiaceae)	Aerial part (EtOH 70%)	ACh, KCl in rat ileum	EO	[20]
**3** Geranyl formate	*Anthemis mauritiana* (Compositae)	Flower (Distillation)	Ca^2+^, carbachol, KCl in rabbit and rat jejunum	EO	[21]
**4** Geranyl acetate	*Nepeta cataria* (Lamiaceae)	Leaf (Aqueous)	Carbachol, KCl in guinea pig trachea and rabbit jejunum	EO	[22]
**5** Geraniol	*Rosa damascene* (Rosaceae)	Flower (hydrodistillation)	ACh, KCl, electrical field stimulation in rat ileum	IC	[23]
**6** Citronellol	*Rosa damascene* (Rosaceae)	Flower (hydrodistillation)	ACh, KCl, electrical field stimulation in rat ileum	IC	[23]
**7** (±)-*α*-Phellandrene	*Zingiber officinale* (Zingiberaceae)	Rhizome (MeOH)	Serotonin in rat ileum	EO	[24]
**8** (±)-*β*-Phellandrene	*Croton sonderianus* (Euphorbiaceae)	Leaf (Distillation)	ACh, KCl in rat tracheal smooth muscle	EO	[25]
**9** Terpinolene	*Zingiber officinale* (Zingiberaceae)	Rhizome (MeOH)	Serotonin in rat ileum	EO	[24]
**10** D-(+)-Limonene	*Zingiber roseum* (Zingiberaceae)	Fresh seeds (Hydrodistilled with diethyl ether)	Carbachol, KCl in rat duodenal smooth muscle	EO	[26]
	*Mentha x villosa *(Lamiaceae)	Leaf infusion (MeOH)	KCl in guinea pig ileum	IC	[27]
	*Dracocephalum kotschyi *(Lamiaceae)	Aerial part (Hydrodistillation)	ACh, electrical field stimulation, KCl in rat ileum	EO	[28]
**11** *γ*-Terpinene	*Acalypha phleoides* (Euphorbiaceae)	Aerial part infusion MeOH-CHCl_3_ (1:1)	ACh, BaCl_2_, H, S in guinea pig ileum and rabbit jejunum	IC	[29]
**12** Thymoquinone	*Nigella sativa* (Ranunculaceae)	Seed infusion (Aqueous)	BaCl_2_, carbachol, leukotriene in rat trachea	IC	[30]
**13** (*R*)-(+)-Pulegone	*Calamintha glandulosa* (Lamiaceae)	Aerial parts infusion (Diethyl ether)	KCl in rat ileum	IC	[31]
	*Mentha x villosa* (Lamiaceae)	Leaf infusion (MeOH)	KCl in guinea pig ileum	IC	[27]
**14** (-)-Menthol	*Mentha piperita *(Lamiaceae)	Leaf and flower infusion (EtOH)	S in rat ileum	IC	[32]
**15** dl-*α*-Terpineol	*Casimiroa pringlei *(Rutaceae)	Aerial part infusion (Ethylic ether)	KCl in rat uterine smooth muscle	IC	[33]
	*Zingiber roseum *(Zingiberaceae)	Fresh seeds (Hydrodistilled with diethyl ether)	Carbachol, KCl in rat duodenal smooth muscle	EO	[26]
	*Dracocephalum kotschyi *(Lamiaceae)	Aerial part (Hydrodistillation)	ACh, electrical field stimulation, KCl in rat ileum	EO	[28]
**16** (-)-Piperitone	*Casimiroa pringlei *(Rutaceae)	Aerial part infusion (Ethylic ether)	KCl in rat uterine smooth muscle	IC	[33]
**17** (+)-Rotundifolone	*Mentha x villosa *(Lamiaceae)	Leaf infusion (MeOH)	KCl in guinea pig ileum	IC	[27]
**18** (*R*)-(-)-Carvone	*Mentha x villosa *(Lamiaceae)	Leaf infusion (MeOH)	KCl in guinea pig ileum	IC	[27]
**19** (*R*,*R*,*R*)-Carvone-1,2-oxide)	*Mentha x villosa *(Lamiaceae)	Leaf infusion (MeOH)	KCl in guinea pig ileum	IC	[27]
**20** (*S*)-(+)-Carvone	*Mentha x villosa *(Lamiaceae)	Leaf infusion (MeOH)	KCl in guinea pig ileum	IC	[27]
**21** 1,8-Cineole	*Ocimum gratissimum *(Lamiaceae)	Leaf infusion (MeOH)	ACh, KCl in guinea pig ileum	IC	[34]
	*Nepeta cataria *(Lamiaceae)	Leaf infusion (Aqueous)	Carbachol, KCl in guinea pig trachea and rabbit jejunum	EO	[22]
	*Casimiroa pringlei *(Rutaceae)	Aerial part infusion (Ethylic ether)	KCl in rat uterine smooth muscle	IC	[33]
**22** *p*-Cymene	*Lippia graveolens* (Verbenaceae)	Leaf infusion (Distillation)	Carbachol, H in guinea pig ileum	IC	[35]
	*Zingiber roseum *(Zingiberaceae)	Fresh seeds (Hydrodistilled with diethyl ether)	Carbachol, KCl in rat duodenal smooth muscle	EO	[26]
	*Poliomintha longiflora* (Lamiaceae)	Leaves stem infusion (Distillation)	Carbachol, H in guinea pig ileum	IC	[35]
**23** Carvacrol	*Origanum acutidens* (Lamiaceae)	Leaf, stem and flower infusion (MeOH)	Spontaneous contraction in rat ileum	EO	[36]
	*Thymus vulgaris* (Lamiaceae)	Whole plants (Ethanol)	ACh, BaCl2, KCl in rat trachea and ileum	IC	[37]
**24** Thymol	*Acalypha phleoides* (Euphorbiaceae)	Aerial part infusion [MeOH-CHCl_3_ (1:1)]	ACh, BaCl_2_, H, KCl, S in guinea pig ileum and rabbit jejunum	IC	[29]
	*Thymus vulgaris* (Lamiaceae)	Whole plants (Ethanol)	ACh, BaCl2, KCl in rat trachea and ileum	IC	[37]
**25** Thujane or Sabinane	*Anthemis mauritiana* (Asteraceae)	Flower infusion (Aqueous)	Carbachol, KCl in rabbit jejunal smooth muscle	EO	[21]
**26** (±)-Camphor	*Acalypha phleoides* (Euphorbiaceae)	Aerial part infusion [MeOH-CHCl_3_ (1:1)]	ACh, BaCl_2_, H, KCl, S in guinea pig ileum and rabbit jejunum	IC	[29]
	*Lippia dulcis *(Verbenaceae)	Leaf infusion (Steam distillation)	Carbachol, H in porcine bronchi	EO	[38]
**27** (+)-*α*-Pinene	*Anthemis mauritiana* (Asteraceae)	Flower infusion (Aqueous)	Carbachol, KCl in rabbit jejunal smooth muscle	EO	[21]
	*Nepeta cataria* (Lamiaceae)	Leaf infusion (Aqueous)	Carbachol, KCl in guinea pig trachea and rabbit jejunum	EO	[22]
	*Plectranthus barbatus* (Lamiaceae)	Leaf infusion (MeOH)	ACh, BaCl_2_, H, KCl in guinea pig ileum	EO	[17]
**28** (-)-*α*-Pinene	*Dissotis rotundifolia* (Melastomataceae)	Leaf infusion (EtOH)	Carbachol in mouse intestinal motility	E	[39]
	*Eucalyptus tereticornis* (Myrtaceae)	Commercial	ACh, KCl in rat trachea	EO	[40]
	*Zingiber roseum *(Zingiberaceae)	Fresh seeds (Hydrodistilled with diethyl ether)	Carbachol, KCl in rat duodenal smooth muscle	EO	[26]
**29** (+)-*β*-Pinene	*Ferula gummosa* (Apiaceae)	Resin infusion (Hydroalcoholic, ether, MeOH)	ACh, KCl in rat ileum	IC	[41]
	*Zingiber officinale* (Zingiberaceae)	Rhizome infusion (MeOH)	S in rat ileum	EO	[24]
	*Zingiber roseum* (Zingiberaceae)	Fresh seeds (Hydrodistilled with diethyl ether)	Carbachol, KCl in rat duodenal smooth muscle	EO	[26]
**30** Cantleyine	*Strychnos trinervis* (Loganiaceae)	Root bark (EtOAc)	Carbachol, H, KCl in guinea pig trachea	IC	[42]
**31** Penstemonoside	*Parentucellia latifolia* (Scrophulariaceae)	Whole plant infusion (Butanol)	ACh, CaCl_2_, KCl in rat uterus	IC	[43]
**32** Aucubine or aucuboside	*Parentucellia latifolia* (Scrophulariaceae)	Whole plant infusion (Butanol)	ACh, CaCl_2_, KCl in rat uterus	IC	[43]
**33** 2′-*O*-Acetyldihydropenstemide	*Viburnum prunifolium* (Caprifoliaceae)	Root and stem bark infusion (MeOH)	Carbachol in rabbit jejunum and guinea pig trachea	E	[44]
**34** 2′-*O*-*trans*-*p*-Coumaroyl-dihydropenstemide	*Viburnum prunifolium* (Caprifoliaceae)	Root and stem bark infusion (MeOH)	Carbachol in rabbit jejunum and guinea pig trachea	E	[44]
**35** 2′-O-Acetylpatrinoside	*Viburnum prunifolium* (Caprifoliaceae)	Root and stem bark infusion (MeOH)	Carbachol in rabbit jejunum and guinea pig trachea	E	[44]
**36** Patrinoside	*Viburnum prunifolium* (Caprifoliaceae)	Root and stem bark infusion (MeOH)	Carbachol in rabbit jejunum and guinea pig trachea	E	[44]
**37** Valtriate or Valepotriate	*Valeriana procera *(Valerianeaceae)	Root infusion (EtOH)	BaCl_2_, carbachol, KCl in guinea pig ileum and stomach	IC	[45]
**38** Isovaltrate or Isovaltratum	*Valeriana procera *(Valerianeaceae)	Root infusion (EtOH)	BaCl_2_, carbachol, KCl in guinea pig ileum and stomach	IC	[45]
**39** Epoxygaertneroside	*Morinda morindoides *(Rubiaceae)	Leaf infusion (Aqueous)	ACh, KCl in guinea pig ileum	IC	[46]
**40** Gaertneroside	*Morinda morindoides *(Rubiaceae)	Leaf infusion (Aqueous)	ACh, KCl in guinea pig ileum	IC	[46]
**41** Catalpinoside or Catapol	*Parentucellia latifolia* (Scrophulariaceae)	Whole plant infusion (Butanol)	ACh, CaCl_2_, KCl in rat uterus	IC	[43]

*Sesquiterpenes *					
**43** (±)-Hernandulcin	*Lippia dulcis *(Verbenaceae)	Leaf infusion (Steam distillation)	Carbachol, H in porcine bronchi	EO	[38]
**43** Humulene or *α*-Caryophyllene	*Nepeta cataria* (Lamiaceae)	Leaf infusion (Aqueous)	Carbachol, KCl, in guinea pig trachea and rabbit jejunum	EO	[22]
**44** *β*-Caryophyllene epoxide	*Conyza filaginoides* (Asteraceae)	Leaf infusion [CHCl_3_:MeOH (1:1)]	Spontaneous contraction in rat ileum	IC	[47]
	*Croton sonderianus *(Euphorbiaceae)	Leaf infusion (Steam distillation)	ACh, KCl in rat tracheal smooth muscle	EO	[25]
**45** *β*-Caryophyllene	*Croton sonderianus *(Euphorbiaceae)	Leaf infusion (Steam distillation)	ACh, KCl in rat tracheal smooth muscle	EO	[25]
	*Conyza filaginoides* (Asteraceae)	Leaf infusion [CHCl_3_:MeOH (1:1)]	Spontaneous contraction in rat ileum	IC	[47]
	*Plectranthus barbatus *(Lamiaceae)	Leaf infusion (MeOH)	ACh, BaCl_2_, H, KCl in guinea pig ileum	EO	[17]
	*Pterodon polygalaeflorus* (Fabaceae)	Seed (Steam distillation)	ACh, KCl in rat ileum smooth muscle	IC	[48]
**46** Bicyclogermacrene or Lepidozene	*Croton sonderianus *(Euphorbiaceae)	Leaf infusion (Steam distillation)	ACh, KCl in rat tracheal smooth muscle	EO	[25]
**47** (+)-Capsidiol	*Nicotiana silvestri *(Solanaceae)	Leaf infusion (EtOAc)	ACh, BaCl_2_, bradykinin, carbachol in guinea pig ileum and trachea	IC	[49]
**48** S-Petasin	*Petasites formosanus* (Compositae)	Aerial parts (EtOH)	CaCl_2_, carbachol, H, KCl in guinea pig trachea	IC	[50]
**49** (+)-Isopetasin	*Petasites formosanus* (Compositae)	Aerial parts (EtOH)	CaCl_2_, carbachol, H, KCl in guinea pig trachea	IC	[50]
**50** Valeranone o Jatamansone	*Valeriana procera *(Valerianeaceae)	Root infusion (EtOH)	BaCl_2_, carbachol, KCl in guinea pig ileum and stomach	IC	[45]
**51** Chamazulene	*Matricaria recutita *(Asteraceae)	Plant infusion (Aqueous)	Human platelet	E	[51]
**52** Spathulenol	*Croton sonderianus *(Euphorbiaceae)	Leaf infusion (Steam distillation)	ACh, KCl in rat tracheal smooth muscle	EO	[25]
	*Lepechinia caulescens* (Lamiaceae)	Leaf infusion (Hexane)	KCl in rat uterus	IC	[52]
**53** Cynaropicrin	*Cynara scolymus *(Asteraceae)	Leaf and flower infusion (MeOH 70%)	ACh in guinea pig ileum	IC	[53]
**54** Cedrenol	*Anthemis mauritiana *(Asteraceae)	Flower infusion (Aqueous)	Carbachol, KCl in rabbit jejunal smooth muscle	EO	[21]
**55** (+)-Bakkenolide A	*Hertia cheirifolia* (Asteraceae)	Aerial parts (MeOH)	ACh, BaCl_2_ in rat duodenum	IC	[54]
**56** Himachalol	*Cedrus deodara* (Pinaceae)	Wood infusion	ACh, BaCl_2_, H, nicotine, S in guinea pig ileum and seminal vesicle, rabbit jejunum and rat uterus	IC	[55]
**57** (E)-Damascenone	*Ipomoea pes-caprae* (Convolvulaceae)	Leaf infusion (Aqueous)	H in guinea pig ileal smooth muscle	IC	[56]
**58** (-)-Isogermacrene D	*Artemisia vulgaris* (Compositae)	Stem and leaf infusion (Aqueous)	guinea pig ileum		[57]
**59** Ezoalantonin	*Artemisia vulgaris *(Compositae)	Leaf (CHCl_3_)	H, PMA, S in guinea pig ileum and trachea	IC	[57]
**60 **Costunolide	*Radix aucklandiae *(Asteraceae)	Rhizome (MeOH)	ACh, KCl, S in rat jejunum	IC	[58]
**61 **Dehydrocostuslactone	*Radix aucklandiae *(Asteraceae)	Rhizome (MeOH)	ACh, KCl, S in rat jejunum	IC	[58]

*Diterpenes *					
**62 **E-Phytol	*Ipomoea pes-caprae* (Convolvulaceae)	Leaf infusion (Aqueous)	H in guinea pig ileal smooth muscle	IC	[56]
**63 **3*α*-Angeloyloxy-2*α*-hydroxy-13,14*Z*-dehydrocativic acid	*Brickellia paniculata* (Compositae)	Leaf infusion (MeOH)	KCl in rat myometrial tissue	IC	[59]
**64 **15-Epicyllenin A	*Marrubium globosum *ssp*. libanoticum *(Lamiaceae)	Aerial part infusion (MeOH)	ACh in mouse ileum	IC	[60]
**65 **Cyllenin A	*Marrubium globosum *ssp*. libanoticum *(Lamiaceae)	Aerial part infusion (MeOH)	ACh in mouse ileum	IC	[60]
**66 **Marrulibacetal	*Marrubium globosum *ssp*. libanoticum *(Lamiaceae)	Aerial part infusion (MeOH)	ACh in mouse ileum	IC	[60]
**67 **(13*R*)-9*α*,13*α*-epoxylabda-6*β*(19),16(15)-diol dilactone	*Marrubium globosum *ssp*. libanoticum *(Lamiaceae)	Aerial part infusion (MeOH)	ACh in mouse ileum	IC	[60]
**68 **Marrubin	*Marrubium vulgare *(Lamiaceae)	Aerial parts (Aqueous)	KCl in rat aorta	IC	[61]
**69 **Marrubenol or Marrubiol	*Marrubium vulgare *(Lamiaceae)	Aerial parts (Aqueous)	KCl in rat aorta	IC	[61]
**70 **Marrulanic acid	*Marrubium globosum *ssp*. libanoticum *(Lamiaceae)	Aerial part infusion (MeOH)	ACh in mouse ileum	IC	[60]
**71 **Marrulactone	*Marrubium globosum *ssp*. libanoticum *(Lamiaceae)	Aerial part infusion (MeOH)	ACh in mouse ileum	IC	[60]
**72 **(+)-Dehydroabietic acid	*Lepechinia caulescens* (Lamiaceae)	Leaf infusion (Hexane)	KCl in rat uterus	IC	[52]
**73 **9*β*-Hydroxydehydroabietyl alcohol	*Lepechinia caulescens* (Lamiaceae)	Leaf infusion (Hexane)	KCl in rat uterus	IC	[52]
**74 **9*α*,13*α*-Epidioxyabiet-8(14)-en-18-oic acid methyl ester	*Lepechinia caulescens* (Lamiaceae)	Leaf infusion (Hexane)	KCl in rat uterus	IC	[52]
**75 **4-epi-Hyalic acid	*Croton argyrophylloides *(Euphorbiaceae)	Bark infusion (MeOH)	ACh, KCl in rat tracheal smooth muscle	IC	[62]
**76 **Pimaradienoic acid or Continentalic acid	*Viguiera arenaria* (Asteraceae)	Root infusion (CH_2_Cl_2_)	ACh, KCl in rat carotid artery	IC	[63]
**77 **8(14),15-Sandaracopimaradiene-7*α*,18-diol	*Tetradenia riparia* (Lamiaceae)	Leaf infusion (CHCl_3_)	BaCl_2_, H, methacholine in guinea pig ileum	IC	[64]
**78 **3,4-Secoisopimara-4(18),7,15-triene-3-oic acid	*Salvia cinnabarina *(Lamiaceae)	Aerial parts (EtOH)	ACh, BaCl_2_, H in guinea pig ileum	IC	[65]
**79 **ent-Kaurenoic acid	*Viguiera arenaria* (Asteraceae)	Root infusion (CH_2_Cl_2_)	ACh, KCl in rat carotid artery	IC	[63]
	*Viguiera hypargyrea* (Asteraceae)	Root infusion (Hexane)	Spontaneous contraction in guinea pig ileum	IC	[66]
**80 **Beyerenic acid or Monogynoic acid	*Viguiera hypargyrea* (Asteraceae)	Root infusion (Hexane)	Spontaneous contraction in guinea pig ileum	IC	[66]
**81 **ent-7*α*-Acetoxytrachyloban-18-oic acid	*Xylopia langsdorfiana *(Annonaceae)	Stem infusion (EtOH 95%)	BaCl_2_, H, KCl in guinea pig ileum	IC	[67]
**82 **ent-7*α* -hydroxytrachyloban-18-oic acid	*Xylopia langsdorfiana *(Annonaceae)	Stem infusion (EtOH 95%)	BaCl_2_, H, KCl in guinea pig ileum	IC	[67]
**83 **Phorbol 12-acetate-13-tiglate	*Crotonis tiglium* (Euphorbiaceae)	Fruit (MeOH)	Spontaneous contraction in rabbit jejunum	E	[68]
**84 **3,7,10,14,15-pentaacetyl-5-butanoyl-13,17-epoxy-8-myrsinene	*Pycnocycla spinosa* (Umbelliferae)	Aerial parts (MeOH)	KCl in rat illeum	IC	[69]

*Triterpenoids*					
**85 **Agapanthagenin 3-O-*β*-D-glucopyranoside	*Allium elburzense *(Alliaceae)	Flower and bulb infusion (Hexane)	H in guinea pig ileum	IC	[70]
**86 **Agapanthagenin	*Allium elburzense *(Alliaceae)	Flower and bulb infusion (Hexane)	H in guinea pig ileum	IC	[70]
**87** *β*-sitosterol	*Eucalyptus camaldulensis* (Myrtaceae)	Leaf infusion (EtOAc)	KCl, spontaneous contraction in rabbit jejunum	IC	[71]
**88 ** *β*-sitosterol 3-*O*-*β*-D-glucopyranoside	*Eucalyptus camaldulensis* (Myrtaceae)	Leaf infusion (EtOAc)	KCl, spontaneous contraction in rabbit jejunum	IC	[71]
**89 ** *α*-Spinasteryl *β*-D-glucoside	*Conyza filaginoides* (Asteraceae)	Leaf infusion [CHCl_3_:MeOH (1:1)]	Spontaneous contraction in rat ileum	IC	[47]
**90 **Tropeoside B1 and B2	*Allium cepa*(Alliaceae)	Bulbs [CHCl_3_:MeOH (9:1)]	ACh, H in guinea pig ileum	IC	[72]
**91 **Tropeoside A1 and A2	*Allium cepa*(Alliaceae)	Bulbs [CHCl_3_:MeOH (9:1)]	ACh, H in guinea pig ileum	IC	[72]
**92 **Elburzensoside A1 and A2	*Allium elburzense *(Alliaceae)	Flower and bulb infusion (Hexane)	H in guinea pig ileum	IC	[70]
**93 **Elburzensoside C1 and C2	*Allium elburzense *(Alliaceae)	Flower and bulb infusion (Hexane)	H in guinea pig ileum	IC	[70]
**94 **Galphimin A	*Galphimia glauca* (Malpighiaceae)	Leaf infusion (MeOH)	Electrical-induced contraction in guinea pig ileum	IC	[73]
**95 **Galphimin B	*Galphimia glauca* (Malpighiaceae)	Leaf infusion (MeOH)	Electrical-induced contraction in guinea pig ileum	IC	[73]
**96 **Galphimin C	*Galphimia glauca* (Malpighiaceae)	Leaf infusion (MeOH)	Electrical-induced contraction in guinea pig ileum	IC	[73]
**97 **Galphimin E	*Galphimia glauca* (Malpighiaceae)	Leaf infusion (MeOH)	Electrical-induced contraction in guinea pig ileum	IC	[73]
**98 **Galphimin F	*Galphimia glauca* (Malpighiaceae)	Leaf infusion (MeOH)	Electrical-induced contraction in guinea pig ileum	IC	[73]
**99 **Handianol	*Herissanthia tiubae* (Malvaceae)	Leaf infusion (EtOH)	Carbachol, H, KCl in guinea pig ileum and trachea, and rat aorta	IC	[74]
**100 **Cycloartanol	*Herissanthia tiubae* (Malvaceae)	Leaf infusion (EtOH)	Carbachol, H, KCl in guinea-pig ileum, trachea and rat aorta	IC	[74]
**101 **Taraxasteryl acetate	*Brickellia veronicifolia* (Asteraceae)	Aerial parts [CH_2_Cl_2_:MeOH (1:1)]	Gastrointestinal motility test in mouse	E	[75]
**102 **Pomolic acid or Benthamic acid or Randialic acid A	*Licania pittieri* (Rosaceae)	Leaf infusion (EtOH)	Carbachol, KCl in rat aorta	IC	[76]
**103 **Ursolic acid	*Agastache mexicana *(Lamiaceae)	Aerial part (MeOH)	ACh, KCl in guinea pig ileum	IC	[77]
**104 **Ehretiolide	*Eucalyptus camaldulensis* (Myrtaceae)	Leaf infusion (EtOAc)	KCl, spontaneous contraction in rabbit jejunum	IC	[78]
**105 **Ehretiolide acetate	*Eucalyptus camaldulensis* (Myrtaceae)	Leaf infusion (EtOAc)	KCl, spontaneous contraction in rabbit jejunum	IC	[78]
**106 **Camaldulin	*Eucalyptus camaldulensis* (Myrtaceae)	Leaf infusion (EtOAc)	KCl, spontaneous contraction in rabbit jejunum	IC	[71]
**107 **Zygophyloside N	*Zygophyllum gaetulum *(Zygophyllaceae)	Root infusion (MeOH)	Electrically-induced contractions of isolated guinea pig ileum	E	[79]
**108 **Erythrodiol	*Conyza filaginoides* (Asteraceae)	Leaf infusion [CHCl_3_:MeOH (1:1)]	Spontaneous contraction in rat ileum	IC	[47]
**109 **3-*β*-tridecanoyloxy-28-hydroxyolean-12-ene	*Conyza filaginoides *(Asteraceae)	Leaf infusion [CHCl_3_:MeOH (1:1)]	Spontaneous contraction in rat ileum	IC	[47]
**110 **3-*β-*Hydroxyolean-9(11),12-dien-28-oic acid	*Eucalyptus camaldulensis* (Myrtaceae)	Leaf infusion (EtOAc)	KCl, spontaneous contraction in rabbit jejunum	IC	[78]
**111 **4-epi-Hederagenin	*Hedera helix *(Araliaceae)	Leaf infusion (EtOH)	ACh in guinea pig ileum	IC	[80]
**112 **Hederacoside C	*Hedera helix *(Araliaceae)	Leaf infusion (EtOH)	ACh in guinea pig ileum	IC	[80]
**113 **Betulinic acid	*Eucalyptus camaldulensis* (Myrtaceae)	Leaf infusion (EtOAc)	KCl, spontaneous contraction in rabbit jejunum	IC	[78]
**114 ** *α*-Amyrin acetate	*Tylophora hirsuta* (Asclepiadaceae)	Aerial parts (MeOH)	KCl in rabbit jejunum	IC	[81]

*Phloroglucinol derivatives*					
**115 **Hyperforin	*Hypericum perforatum* (Hypericaceae)	Aerial parts (EtOH 70%)	KCl in rabbit jejunum	IC	[82]
**116 **Hypericin	*Hypericum perforatum* (Hypericaceae)	Aerial parts (EtOH 70%)	KCl in rabbit jejunum	IC	[82]

*Coumarins*					
**117 **Scopoletin	*Brunfelsia hopeana* (Solanaceae)	Root infusion (EtOH)	Phenylephrine, KCl, PGF2, serotonin in rat aorta	IC	[83]
**118 **Todannone	*Toddalia asiatica *var. floribunda (Rutaceae)	Aerial parts (EtOH 95%)	ACh, BaCl_2_, H, nicotine in guinea pig ileum	IC	[84]
**119 **(2S*∗*,3R*∗*)-2-[(3E)-4,8-dimethylnona-3,7-dien-1-yl]-2,3-dihydro-7-hydroxy-2,3-dimethylfuro[3,2c] coumarin	*Ferula heuffelii* (Apiaceae)	Underground part (CHCl_3_)	ACh, KCl in rat ileum	IC	[85]
**120 **Osthole	*Prangos ferulacea* (Apiaceae)	Root (Acetone)	ACh, KCl, electric field stimulation in rat ileum	IC	[86]
**121 **Angelicin	*Heracleum thomsoni* (Apiaceae)	Aerial part infusion (EtOH)	ACh, BaCl_2_, H, S in cat ureter, guinea pig bile duct and trachea, monkey gall bladder, rabbit jejunum, and rat uterus	IC	[87]
**122 **Glycycoumarin	*Glycyrrhizae radix* (Leguminosae)	Root infusion (Aqueous)	A23187, BaCl_2_, carbachol, KCl in mouse jejunum	IC	[88]
	*Glycyrrhiza ularensis *(Leguminosae)	Root infusion (Aqueous)	Carbachol in mouse jejunum	E	[89]

*Chalcones*					
**123 **Davidigenin	*Mascarenhasia arborescens* (Apocynaceae)	Leaf and stem infusion (MeOH)	ACh, H in guinea pig and rat duodenum	IC	[90]
**124 **Isoliquiritigenin	*Glycyrrhiza glabra *(Leguminosae)	Root infusion (Aqueous)	ACh, KCl, O, spontaneous contraction in rat uterus	IC	[91]
	*Glycyrrhiza ularensis *(Leguminosae)	Root infusion (Aqueous) (Aqueous)	BaCl_2_, carbachol, KCl in mouse jejunum,ileum and rectum	IC	[92]
**125 **Licochalcone A	*Glycyrrhiza inflata* (Leguminosae)	Root infusion (Aqueous)	A23187, BaCl_2_, carbachol, KCl in mouse jejunum	IC	[93]

*Flavonoids *					
**126 **(-)-Pinostrobin	*Conyza filaginoides* (Asteraceae)	Leaf infusion [CHCl_3_:MeOH (1:1)]	Spontaneous contraction in rat ileum	IC	[47]
**127 **(-)-(S)-Sakuranetin	*Dodonaea viscosa* (Sapindaceae)	Leaf infusion [CHCl_3_:MeOH (1:1)]	ACh, BaCl_2_, H in rat uterus	IC	[94]
**128 **(±)-Sternbin	*Artemisia monosperma* (Compositae)	Aerial part (EtOH)	ACh, O in rat ileum, pulmonary artery, urinary bladder, trachea, and uterus	IC	[95]
**129 **Ouratea catechin	*Maytenus rigida* (Celastraceae)	Stem bark (EtOH)	BaCl_2_, carbachol, KCl, H in guinea pig ileum	IC	[96]
**130 **Apegenin	*Achillea millefolium* (Asteraceae)	Whole plant infusion (MeOH 40%)	ACh, CaCl_2_, H, PE, S in rat ileum	IC	[97]
**131 **Buddleoflavonol or Linarigenin	*Agastache mexicana *(Lamiaceae)	Aerial part (MeOH)	ACh, KCl in guinea pig ileum	IC	[77]
**132 **Luteolin	*Achillea millefolium* (Asteraceae)	Whole plant infusion (MeOH 40%)	ACh, CaCl_2_, H, PE, S in rat ileum	IC	[97]
	*Artemisia copa* (Compositae)	Aerial parts (Aqueous)	KCl, PE, S in rat aorta	E	[98]
	*Plantago lanceolata* (Plantaginaceae)	Aerial part (EtOH)	ACh, BaCl_2_, H, KCl in guinea pig ileum and trachea	IC	[99]
	*Thymus vulgaris *(Lamiaceae)	Leaf and flower (EtOH)	ACh, BaCl_2_, carbachol, H in guinea pig ileum and trachea, and rat vas deferens	IC	[100]
**133 **Scutellarein 6-*β*-D-glucoside (isovitexin)	*Aloysia citridora* (Verbenaceae)	Leaf infusion (Aqueous)	ACh, CaCl_2_, KCl in rat duodenum	IC	[101]
**134 **Vitexin	*Aloysia citridora* (Verbenaceae)	Leaf infusion (Aqueous)	ACh, CaCl_2_, KCl in rat duodenum	IC	[101]
	*Aspalathus linearis *(Fabaceae)	Commercial (Aqueous)	KCl in rabbit jejunum	IC	[102]
**135 **Xanthomycrol	*Brickellia paniculata* (Compositae)	Leaf infusion (MeOH)	KCl, O in rat uterus	IC	[59]
**136 **Demethoxycentaureidin	*Piptadenia stipulacea* (Leguminosae)	Aerial parts, (CHCl_3_)	Carbachol, H, O, in guinea pig ileum and trachea, rat aorta and uterus	IC	[103]
**137 **Gnaphaliin B	*Gnaphalium liebmannii* (Asteraceae)	Aerial parts (Hexane)	ACh, carbachol in guinea pig trachea	IC	[104]
**138 **Kaempferol or Kaempherol	*Hedera helix *(Araliaceae)	Aerial parts (EtOH 30%)	ACh in guinea pig ileum	IC	[80]
**139 **Gnaphaliin A	*Gnaphalium liebmannii* (Asteraceae)	Aerial parts (Hexane)	ACh, carbachol in guinea pig trachea	IC	[104]
**140 **Quercetin	*Achillea millefolium* (Asteraceae)	Whole plant infusion (MeOH 40%)	ACh, CaCl_2_, H, PE, serotonin in rat ileum	IC	[97]
	*Psidium guajava *(Myrtaceae)	Leaf extract (MeOH)	Peristalsis in guinea pig ileum	IC	[105]
	*Drosera madascariensis* (Droseraceae)	Leaf extract (EtOH 70%)	Carbachol, H, PGF2 in guinea pig ileum and trachea	IC	[106]
	*Drosera rotundifolia *(Droseraceae)	Aerial parts (EtOH 70%)	Carbachol in guine pig ileum	IC	[107]
	*Morinda morindoides *(Rubiaceae)	Leaf extract (Aqueous)	Ac, KCl in guinea pig ileum	IC	[46]
**141 **3-O-Methylquercetin	*Rhamnus nakaharai* (Rhamnaceae)	Stem bark (not reported)	Carbachol, H, KCl in guinea pig trachea	IC	[108]
**142 **3,4′-Dimethylquercetin	*Artemisia abrotanum* (Asteraceae)	Aerial part (MeOH 67%)	Carbachol in guinea pig trachea	IC	[109]
**143 **3,7-Dimethylquercetin	*Artemisia abrotanum* (Asteraceae)	Aerial part (MeOH 67%)	Carbachol in guinea pig trachea	IC	[109]
**144 **Isoquercetin	*Conyza filaginoides* (Asteraceae)	Leaf infusion [CHCl_3_:MeOH (1:1)]	Spontaneous contraction in rat ileum	IC	[47]
	*Hedera helix *(Araliaceae)	Aerial parts (EtOH 30%)	ACh in guinea pig ileum	IC	[80]
	*Drosera rotundifolia *(Droseraceae)	Aerial parts (EtOH 70%)	Carbachol in guinea pig ileum	IC	[107]
	*Drosera madascariensis *(Droseraceae)	Leaf extract (EtOH 70%)	Carbachol, H, PGF2 in guinea pig ileum and trachea	IC	[106]
	*Psidium guajava *(Myrtaceae)	Leaf extract (MeOH)	Peristalsis in guinea pig ileum	IC	[105]
**145 **Quercetin 3-*α*-rhamnoside or Quercitroside	*Psidium guajava *(Myrtaceae)	Leaf extract (MeOH)	Peristalsis in guinea pig ileum	IC	[105]
	*Morinda morindoides *(Rubiaceae)	Leaf extract (Aqueous)	ACh, KCl in guinea pig ileum	IC	[46]
**146 **Quercetin 3-O-*β*-L-arabinoside	*Psidium guajava *(Myrtaceae)	Leaf extract (MeOH)	Peristalsis in guinea pig ileum	IC	[105]
**147 **Quercetin 3-O-*β*-D-galactoside	*Psidium guajava *(Myrtaceae)	Leaf extract (MeOH)	Peristalsis in guinea pig ileum	IC	[105]
	*Drosera madascariensis *(Droseraceae)	Leaf extract (EtOH 70%)	Carbachol, H, PGF2 in guinea pig ileum and trachea	IC	[106]
**148 **Quercetin 3-O-*β*-gentiobioside 3-O-*β*-D-	*Morinda morindoides *(Rubiaceae)	Leaf extract (Aqueous)	ACh, KCl in guinea pig ileum	IC	[46]
Glucopyranosylquercetin	*Drosera rotundifolia *(Droseraceae)	Aerial parts (EtOH 70%)	Carbachol in guinea pig ileum	EO	[107]
**149 **Centaureidin	*Artemisia abrotanum* (Asteraceae)	Aerial part (MeOH 67%)	Carbachol in guinea pig trachea	IC	[109]
**150 **Casticin or Vitexicarpin	*Artemisia abrotanum* (Asteraceae)	Aerial part (MeOH 67%)	Carbachol in guinea pig trachea	IC	[109]
**151 **Prunetol or Sophoricol	*Genista tridentata* (Papilionaceae)	Not reported	AC, electric field stimulation, 6-oxo PGE1 in guinea pig ileum	IC	[110]
**152 **Boeravinone E	*Boerhaavia diffusa* (Nyctaginaceae)	Root infusion (MeOH)	ACh in guinea pig ileum	IC	[111]
**153 **4,6,11-trihydroxy-9-methoxy-10-methyl-6,12-dihydro-5,7-dioxatetraphen-12-one	*Boerhaavia diffusa* (Nyctaginaceae)	Root infusion (MeOH)	ACh in guinea pig ileum	IC	[111]
**154 **Boeravinone G	*Boerhaavia diffusa* (Nyctaginaceae)	Root infusion (MeOH)	ACh in guinea pig ileum	IC	[111]
**155 **(2R,3S,2”R,3”R)-Manniflavonone	*Garcinia buchananii* (Clusiaceae)	Stem bark (EtOH 70%)	Bay K 8644 in mouse ileum	IC	[112]
**156 **Hyperoside	*Hypericum perforatum* (Hypericaceae)	Aerial parts (EtOH 70%)	KCl in rabbit jejunum	IC	[82]
**157 **Chrysoeriol	*Artemisia copa *(Compositae)	Aerial parts (Aqueous)	KCl, PE, S in rat aorta	E	[98]
	*Aspalathus linearis *(Fabaceae)	Commercial (Aqueous)	KCl in rabbit jejunum	IC	[102]
**158 **Spinacetin	*Artemisia copa *(Compositae)	Aerial parts (Aqueous)	KCl, PE, S in rat aorta	E	[98]
**159 **Vicenin 2	*Perilla frutescens *(Lamiaceae)	Commercial (Aqueous)	ACh, BaCl_2_ i rat ileum	IC	[113]
**160 **Orientin	*Aspalathus linearis *(Fabaceae)	Commercial (Aqueous)	KCl in rabbit jejunum	IC	[102]

*Phenylmetanoids*					
**161 **Salicylic acid methyl ether	*Brickellia veronicifolia* (Asteraceae)	Aerial parts [CH_2_Cl_2_:MeOH (1:1)]	Gastrointestinal motility test in mouse	E	[75]
**162 ** *O*-Anisic acid or 6-Methoxysalicylic acid	*Brickellia veronicifolia* (Asteraceae)	Aerial parts [CH_2_Cl_2_:MeOH (1:1)]	Gastrointestinal motility test in mouse	E	[75]
**163 **Protocatechuic acid	*Hedera helix *(Araliaceae)	Aerial parts (EtOH 30%)	ACh in guinea pig ileum	IC	[80]
**164 **Benzyl 2,5-dimethoxybenzoate	*Brickellia veronicifolia *(Asteraceae)	Aerial parts [CH_2_Cl_2_-MeOH (1:1)]	Gastrointestinal motility test in mouse	E	[75]

*Phenylethanoids*					
**165 **O-Methylbalsamide	*Zanthoxylum hyemale* (Rutaceae)	Stem bark infusion (EtOH)	ACh, BaCl_2_ in rat ileum	IC	[114]
**166 **(-)-Tembamide	*Zanthoxylum hyemale* (Rutaceae)	Stem bark infusion (EtOH)	ACh, BaCl_2_ in rat ileum	IC	[114]
**167 **O-Methyltembamide	*Zanthoxylum hyemale* (Rutaceae)	Steam bark infusion (EtOH)	ACh, BaCl_2_ in rat ileum	IC	[114]

*Phenylpropanoids*					
**168 **Eugenol	*Ocimum gratissimum *(Lamiaceae)	Not reported	ACh, KCl in guinea pig ileum	EO	[34]
**169 **Rosemaric acid or Rosemary acid or *trans*-Rosmarinic acid	*Thymus vulgaris* (Lamiaceae)	Commercial	KCl in rat trachea	IC	[100]
**170 ** *trans*-Chlorogenic acid	*Hedera helix *(Araliaceae)	Aerial parts (EtOH 30%)	ACh in guinea pig ileum	IC	[80]
**171 ** *cis*-Chlorogenic acid	*Hedera helix *(Araliaceae)	Aerial parts (EtOH 30%)	ACh in guinea pig ileum	IC	[80]
**172 **3,5-Dicaffeoylquininic acid	*Hedera helix *(Araliaceae)	Aerial parts (EtOH 30%)	ACh in guinea pig ileum	IC	[80]
**173 **Verbascoside	*Plantago lanceolata* (Plantaginaceae)	Aerial part infusion (EtOH 20%)	ACh, BaCl_2_, H, KCl in guinea pig ileum and trachea	E	[99]
**174 **Isoacteoside or Isoverbascoside	*Plantago lanceolata* (Plantaginaceae)	Aerial part infusion (EtOH 20%)	ACh, BaCl_2_, H, KCl in guinea pig ileum and trachea	E	[99]
**175 **Plantamajoside or Plantamoside or Purpureaside A	*Plantago lanceolata* (Plantaginaceae)	Aerial part infusion (EtOH 20%)	ACh, BaCl_2_, H, KCl in guinea pig ileum and trachea	E	[99]
**176 **Lavandulifolioside	*Plantago lanceolata* (Plantaginaceae)	Aerial part infusion (EtOH 20%)	ACh, BaCl_2_, H, KCl in guinea pig ileum and trachea	E	[99]
**177 **Echinacoside	*Cistanche tubulosa *(Orobanchaceae)	No reported (EtOH)	KCl, PE in rat aorta	IC	[115]
**178 **Schisandrin A or Wuweizisu A	*Schisandra chinensis *(Schisandraceae)	Academic	Spontaneous contractions in rat colon	IC	[116]
**179 **Schisandrin B or Wuweizisu B	*Schisandra chinensis *(Schisandraceae)	Fruit decoction (Aqueous)	ACh, KCl, S in guinea pig ileum	IC	[117]
**180 **Schisandrol B	*Schisandra chinensis *(Schisandraceae)	Fruit decoction (Aqueous)	ACh, KCl, S in guinea pig ileum	IC	[117]

*Stilbenoids*					
**181 **Aloifol II or Dendrophenol or Moscatilin	*Nidema boothii* (Orchidaceae)	Whole plant infusion [CH_2_Cl_2_-MeOH 1:1)]	Spontaneous contraction in guinea pig ileum	IC	[118]
**182 **Batatasin III	*Nidema boothii* (Orchidaceae)	Whole plant infusion [CH_2_Cl_2_-MeOH 1:1)]	Spontaneous contraction in guinea pig ileum	IC	[118]
	*Scaphyglottis livida* (Orchidaceae)	Whole plant infusion [CH_2_Cl_2_-MeOH (1:1)]	ACh, BaCl_2_, H in rat ileum	IC	[119]
**183 **4-[2-(3-hydroxy-5-methoxyphenyl)ethyl]-2-methoxyphenol	*Scaphyglottis livida* (Orchidaceae)	Whole plant infusion [CH_2_Cl_2_-MeOH (1:1)]	ACh, BaCl_2_, H in rat ileum	IC	[119]
**184 **Gigantol	*Nidema boothii* (Orchidaceae)	Whole plant infusion [CH_2_Cl_2_-MeOH (1:1)]	Spontaneous contraction in guinea pig ileum	IC	[118]
**185 **Coelonin	*Scaphyglottis livida* (Orchidaceae)	Whole plant infusion [CH_2_Cl_2_-MeOH (1:1)]	ACh, BaCl_2_, H in rat ileum	IC	[119]
**186** Erianthridin	*Maxillaria densa *(Orchidaceae)	Whole plant infusion [CHCl_3_-MeOH (1:1)]	ACh, BaCl_2_, H in rat ileum	IC	[120]
**187** Ephemeranthoquinone	*Nidema boothii* (Orchidaceae)	Whole plant infusion [CH_2_Cl_2_-MeOH (1:1)]	Spontaneous contraction in guinea pig ileum	IC	[118]
**188** Nudol	*Maxillaria densa *(Orchidaceae)	Whole plant infusion [CHCl_3_-MeOH (1:1)]	ACh, BaCl_2_, H in rat ileum	IC	[120]
**189** 3,4- dimethoxyphenanthrene-2,5-diol	*Maxillaria densa *(Orchidaceae)	Whole plant infusion [CHCl_3_-MeOH (1:1)]	ACh, BaCl_2_, H in rat ileum	IC	[120]
**190** Denthyrsinin	*Scaphyglottis livida *(Orchidaceae)	Whole plant infusion [CH_2_Cl_2_-MeOH (1:1)]	ACh, BaCl_2_, H in rat ileum	IC	[119]
**191** Gymnopusin	*Maxillaria densa *(Orchidaceae)	Whole plant infusion [CHCl_3_-MeOH (1:1)]	ACh, BaCl_2_, H in rat ileum	IC	[120]
**192** Fimbriol A	*Maxillaria densa *(Orchidaceae)	Whole plant infusion [CHCl_3_-MeOH (1:1)]	ACh, BaCl_2_, H in rat ileum	IC	[120]

*Curcuminoid*					
**193** (1E,5S,6E)-5-hydroxy-1,7-bis(4-hydroxy-3-methoxyphenyl)-1,6-heptadien-3-one	*Curcuma longa *(Zingiberaceae)	Macerated rhizome (EtOH 70%)	ACh, BaCl_2_, CaCl_2_, H, KCl, O in guinea pig Ileum and rat uterus	IC	[121]

*Benzofurans and Related*					
**194** (+)-Vitisin C	*Vitis* spp. (Vitaceae)	Stem infusion (MeOH)	PE in rabbit aorta	IC	[122]
**195** Butylphthalide	*Ligusticum wallichii* (Umbelliferae)	Rhizome (hydrodistillation)	CaCl_2_, KCl in rat aorta	EO	[123]
**196** cis-Butylidenephthalide	*Ligusticum wallichii* (Umbelliferae)	Rhizome (hydrodistillation)	CaCl_2_, KCl in rat aorta	EO	[123]
**197** Ligustilide A or cis-Ligustilide	*Ligusticum wallichii* (Umbelliferae)	Rhizome (hydrodistillation)	CaCl_2_, KCl in rat aorta	EO	[123]
**198** 12-acetoxytremetone	*Helichrysum italicum *ssp.* italicum *(Asteraceae)	Flowers (EtOH)	ACh, BaCl_2_ in mouse ileum	IC	[124]
**199** 1-[(2R)-2-(3-hydroxyprop-1-en-2-yl)-2,3-dihydro-1-benzofuran-5-yl]ethan-1-one	*Helichrysum italicum *ssp.* italicum *(Asteraceae)	Flowers (EtOH)	ACh, BaCl_2_ in mouse ileum	IC	[124]

*Alkaloids*					
**200** Indicaxanthin	*Opuntia ficus indica* (Cactaceae)	Fruit pulp infusion (Aqueous)	Carbachol, KCl in mouse ileum	IC	[125]
**201** Papaverine	*Daucus carota *(Apiaceae)	Seed infusion (MeOH 90%)	ACh, BaCl_2_, H, KCl, S, O in dog trachea, guinea pig, rabbit, rat ilea, rat uterus	IC	[126]
**202** Higenamine	*Nandina domestica* (Berberidaceae)	Fruit (Aqueous)	ACh, H, KCl in guinea pig trachea	IC	[127]
**203** Atherosperminine	*Fissistigma glaucescens *(Annonaceae)	Bark (MeOH)	Carbachol, KCl, LTC4, PGF2*α*, U46619 in guinea pig trachea	IC	[128]
**204** (+)-Domestine or (+)-Nantenine	*Platycapnos spicata* (Fumariaceae)	Academic supplier	BaCl_2_, CaCl_2_, KCl, PE, S in rat aorta and atria	IC	[129]
**205** 10-Methylacridone	*Citrus deliciosa *(Rutaceae)	Root juice (MeOH)	Rabbit ileum	IC	[130]
**206** Spermatheridine or liriodenin	*Fissistigma glaucescens* (Annonaceae)	Leaf infusion (MeOH)	Carbachol in canine trachea	IC	[131]
**207** Citpressine I	*Citrus deliciosa *(Rutaceae)	Root juice (MeOH)	Rabbit ileum	IC	[130]
**208** Jatrorhizine or Neprotine	*Berberis aristata* (Berberidaceae)	Institutional supplier	ACh, S, spontaneous contractions in rat ileum	IC	[132]
	*Coptis chinensis *(Ranunculaceae)	Rhizoma (EtOH 70%)	ACh in guinea pig ileum	IC	[133]
**209** Coptisine	*Coptis chinensis *(Ranunculaceae)	Rhizoma (EtOH 70%)	ACh in guinea pig ileum	IC	[133]
**210** Escholine or Thalictrine	*Mahonia aquifolium* (Berberidaceas)	Cortex and fruit infusion	KCl, PE in rat aorta	IC	[134]
**211** (+)-Isothebaine	*Mahonia aquifolium* (Berberidaceas)	Cortex and fruit infusion	KCl, PE in rat aorta	IC	[134]
**212** (+)-Corytuberine	*Mahonia aquifolium* (Berberidaceas)	Cortex and fruit infusion	KCl, PE in rat aorta	IC	[134]
**213** (+)-Isocorydine or Luteanine	*Mahonia aquifolium* (Berberidaceas)	Cortex and fruit infusion	KCl, PE in rat aorta	IC	[134]
**214** (+)-Chelidonine or Stylophorine	*Chelidonium majus* (Papaveraceae)	Commercial supplier	BaCl_2_, carbachol in guinea pig ileum	IC	[135]
**215** (-)-8 beta-(4′-hydroxybenzyl)-2,3-dimethoxyberbin-10-ol	*Aristolochia constricta* (Aristolochiaceae)	Aerial part infusion (MeOH)	ACh, electrical contraction, H in guinea pig ileum	IC	[136]
**216** 3-O-methylconstrictosine	*Aristolochia constricta* (Aristolochiaceae)	Aerial part infusion (MeOH)	ACh, electrical contraction, H in guinea pig ileum	IC	[136]
**217** 3,5-di-O-methylconstrictosine	*Aristolochia constricta* (Aristolochiaceae)	Aerial part infusion (MeOH)	ACh, electrical contraction, H in guinea pig ileum	IC	[136]
**218 **5,6-dihydro-3,5-di-*O*-methylconstrictosine	*Aristolochia constricta* (Aristolochiaceae)	Aerial part infusion (MeOH)	ACh, electrical contraction, H in guinea pig ileum	IC	[136]
**219** 5,6-dihydroconstrictosine	*Aristolochia constricta* (Aristolochiaceae)	Aerial part infusion (MeOH)	ACh, electrical contraction, H in guinea pig ileum	IC	[136]
**220** Constrictosine	*Aristolochia constricta* (Aristolochiaceae)	Aerial part infusion (MeOH)	ACh, electrical contraction, H in guinea pig ileum	IC	[136]
**221** Isojuripidine	*Solanum asterophorum* (Solanaceae)	Leaf infusion (MeOH)	ACh, CaCl_2_, H in guinea pig ileum	IC	[137]
**222** Sarcodine	*Sarcocca saligna* (Buxaceae)	Whole plant (MeOH)	ACh, KCl in guinea pig ileum, rat stomach fundus, rabbit jejunum	IC	[138]
**223** Saracorine or Sarcorine	*Sarcococca saligna* (Buxaceae)	Whole plant infusion (MeOH)	ACh, KCl in rabbit jejunum	IC	[139]
**224** Saracocine	*Sarcocca saligna* (Buxaceae)	Whole plant (MeOH)	ACh, KCl in guinea pig ileum, rat stomach fundus, rabbit jejunum	IC	[138]
**225** Alkaloid C	*Sarcocca saligna *(Buxaceae)	Whole plant (MeOH)	ACh, KCl in guinea pig ileum, rat stomach fundus, rabbit jejunum	IC	[138]
**226** (-)-Pachyaximine A	*Sarcococca saligna* (Buxaceae)	Whole plant infusion (MeOH)	ACh, KCl in rabbit jejunum, KCl	IC	[139]
**227** (-)-(R)-Geibalansine or (-)-R-Geilbalansine	*Zanthoxylum hyemale* (Rutaceae)	Stem bark infusion (EtOH)	ACh, BaCl_2_ in rat ileum	IC	[114]
**228** Hyemaline	*Zanthoxylum hyemale* (Rutaceae)	Stem bark infusion (EtOH)	ACh, BaCl_2_ in rat ileum	IC	[114]
**229** Theophylline	*Fissistigma glaucescens* (Annonaceae)	Leaf infusion (MeOH)	Carbachol in canine trachea	IC	[131]
**230 **Carboxyscotangamine A	*Scopolia tangutica* (Solanaceae)	Root (95% EtOH)	Carbachol in Chinese hamster ovarian cell	IC	[140]
**231 **Scotanamine A	*Scopolia tangutica* (Solanaceae)	Root (95% EtOH)	Carbachol in Chinese hamster ovarian cell	IC	[140]
**232 **Piperine	*Piper nigrum* (Piperaceae)	Fruit (EtOH)	Ileum loop in mice	IC	[141]

*Amines*					
**233 **Scotanamine B	*Scopolia tangutica* (Solanaceae)	Root (95% EtOH)	Carbachol in Chinese hamster ovarian cell	IC	[123]
**234 **Scotanamine C	*Scopolia tangutica* (Solanaceae)	Root (95% EtOH)	Carbachol in Chinese hamster ovarian cell	IC	[140]
**235 **Scotanamine D	*Scopolia tangutica* (Solanaceae)	Root (95% EtOH)	Carbachol in Chinese hamster ovarian cell	IC	[140]
**236 **N^1^-Caffeoyl-N^3^-dihydrocaffeoylspermidine	*Scopolia tangutica* (Solanaceae)	Root (95% EtOH)	Carbachol in Chinese hamster ovarian cell	IC	[140]
**237 **N^1^, N^10^-Bis(dihydrocaffeoyl)spermidine	*Scopolia tangutica* (Solanaceae)	Root (95% EtOH)	Carbachol in Chinese hamster ovarian cell	IC	[140]
**238 **Caffeoylputrescine	*Scopolia tangutica* (Solanaceae)	Root (95% EtOH)	Carbachol in Chinese hamster ovarian cell	IC	[140]

*Isothiocyanates*					
**239** Redskin or Senfoel	*Cruciferous vegetables* (Brassicaceae)	Commercial source	ACh, electrical contraction in mouse ileum	IC	[142]

*Alcohols*					
**240** (3E)-4-(3,4-dimethoxyphenyl)but-3-en-1-ol	Zingiber cassumunar (Zingiberaceae)	Chemically synthesized	O in rat uterus	IC	[143]

*Ketones*					
**241** 2-Decanone	*Ruta chalepensis *(Rutaceae)	Leaf (EtOH 70%)	KCl in rat ileum	E	[144]
**242** 2-Undecanone	*Ruta chalepensis *(Rutaceae)	Leaf (EtOH 70%)	KCl in rat ileum	E	[144]
**243** 2-Tridecanone	*Ruta chalepensis *(Rutaceae)	Leaf (EtOH 70%)	KCl in rat ileum	E	[144]
**244** Latifolone	*Ferula heuffelii* (Apiaceae)	Underground part (CHCl_3_)	ACh, KCl in rat ileum	IC	[85]
**245 **Dshamirone	*Ferula heuffelii* (Apiaceae)	Underground part (CHCl_3_)	ACh, KCl in rat ileum	IC	[85]

*Phenolic compounds*					
**246** 6-(4-hydroxy-3-methoxyphenyl)-hexanonic acid (HMPHA)	*Pycnocycla spinosa *(Umbelliferae)	Aerial parts (MeOH)	KCl in rat ileum	IC	[145]
**247** Isovanillin	*Pycnocycla spinosa *(Umbelliferae)	Aerial parts (MeOH)	KCl in rat ileum	IC	[146]
**248** Iso-acetovanillon	*Pycnocycla spinosa *(Umbelliferae)	Aerial parts (MeOH)	KCl in rat ileum	IC	[146]

IC = isolated compound, E = extract, EO = essential oil, ACh = acetylcholine, O = oxytocin, PMA = *β*-Phenylethyl amsine, PGF = Prostaglandin F2*α*, H = histamine, S = serotonin.

**Table 4:** Synthetic antispasmodic compounds used in medicine.

**Synthetic compound**	**Receptor targeted**	**Main use**
*Alkaloids*		
Chlorzoxazone	Prevents release of histamine	Muscular spasm
Pancuronium	Nicotinic acetylcholine	Muscle relaxant
Riluzole	Sodium channels	Amyotrophic lateral sclerosis
Rocuronium	Antagonist of neuromuscular junction	Muscle relaxant and anaesthesia
Tizanidine	*α*_2_ adrenergic agonist	Muscle relaxant
Vecuronium	Nicotinic acetylcholine	Muscle relaxant and anaesthesia

*Curcuminoids*		
Atracurium	Nicotinic acetylcholine	Muscle relaxant and anaesthesia
Cisatracurium	Nicotinic acetylcholine	Muscle relaxant and anaesthesia
Mivacurium	Nicotinic acetylcholine	Muscle relaxant and anaesthesia

*Methylpropanoid*		
Diazepam	GABA_A_	Anxiety, alcohol withdrawal syndrome, muscle spasms, seizures, and restless legs syndrome
Prograbide	GABA_A+B_	Epilepsy
Orphenadrine		Skeletal muscle relaxant that is used for the treatment of acute muscle aches, pain, or spasms.

*Phenylpropanoids*		
Baclofen	GABA_B_	Spinal cord injury, cerebral palsy, and multiple sclerosis
Idrocilamide	Prevents release of intracellular Ca^2+^	Skeletal muscle relaxant and muscular pain

**Table 5:** Similarities between natural and synthetic compounds.

**Synthetic**	**Natural **
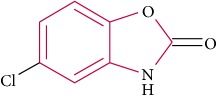	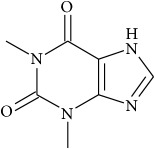
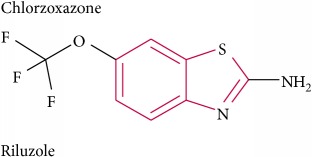	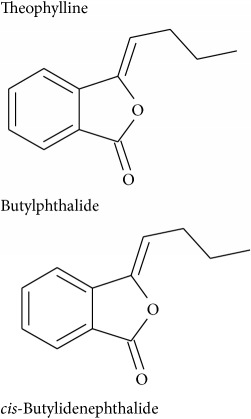

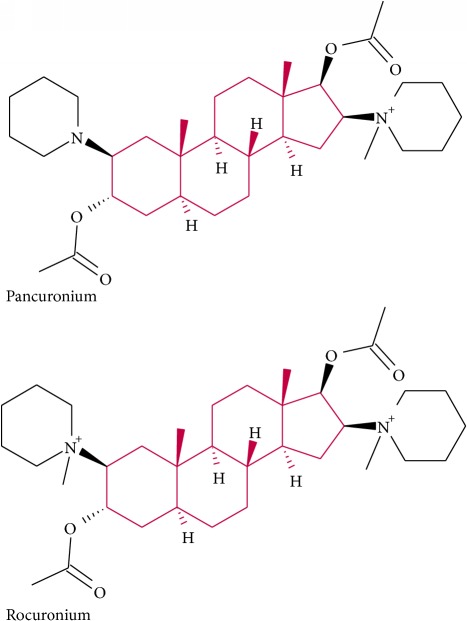	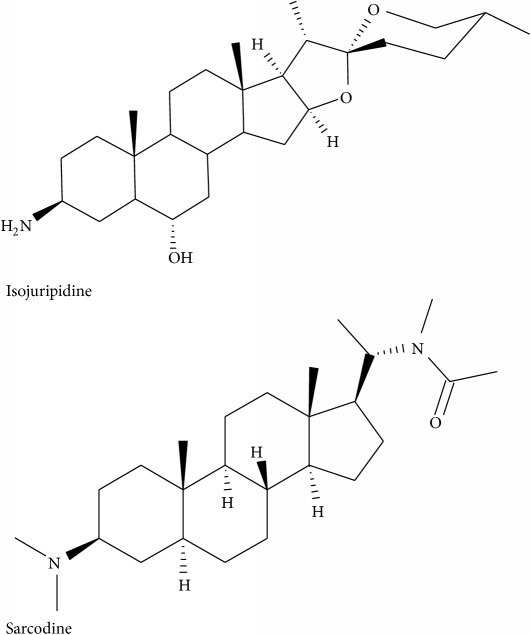

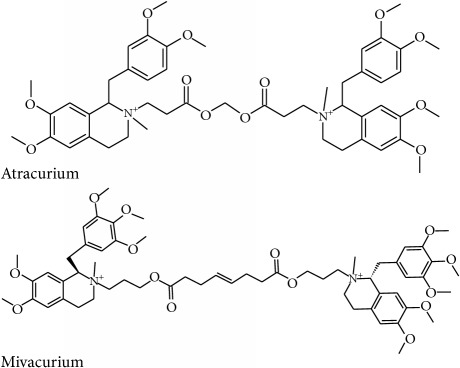	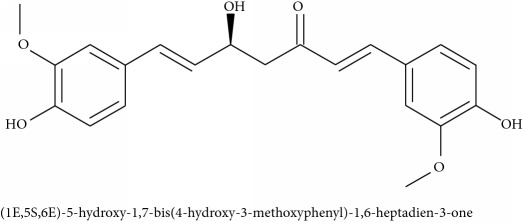

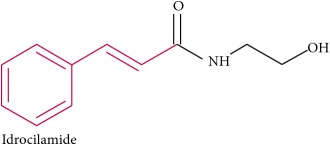	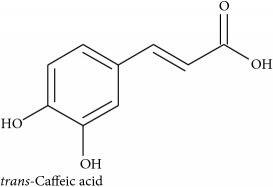

## Supplementary Materials

Supplementary Materials This file contains the structures of the compounds described in the main text.
