# Supplementary material for: Natural Antispasmodics: Source, Stereochemical Configuration, and Biological Activity
Source: Biomed Res Int. 2018 Oct 8;2018:3819714. doi: 10.1155/2018/3819714 (PMC6196993; doi:10.1155/2018/3819714)
Supplement: Supplementary Materials — This file contains the structures of the compounds described in the main text. [file 3819714.f1.docx]

|   **1** |
| --- |
|   **2** |
|   **3** |
|   **4** |
|   **5** |
|   **6** |
|   **7** |
|   **8** |
|   **9** |
|   **10** |
|   **11** |
|   **12** |
|   **13** |
|   **14** |
|   **15** |
|   **16** |
|   **17** |
|   **18** |
|   **19** |
|   **20** |
|   **21** |
|   **22** |
|   **23** |
|   **24** |
|   **25** |
|   **26** |
|   **27** |
|   **28** |
|   **29** |
|   **30** |
|   **31** |
|   **32** |
|   **33** |
|   **34** |
|   **35** |
|   **36** |
|   **37** |
|   **38** |
|   **39** |
|   **40** |
|   **41** |
| **42**  |
|  **43** |
|    **44** |
|  **45** |
| **46**  |
|  **47** |
| **48**  |
| **49**  |
|  **50** |
|    **51** |
|  **52** |
|  **53** |
|  **54** |
|   **55** |
|  **56** |
|   **57** |
|   **58** |
|   **59** |
|   **60** |
|   **61** |
|   **62** |
|   **63** |
|   **64** |
|   **65** |
|   **66** |
|   **67** |
|   **68** |
|   **69** |
|   **70** |
|   **71** |
|   **72** |
|   **73** |
|   **74** |
|   **75** |
|   **76** |
|   **77** |
|   **78** |
|   **79** |
|   **80** |
|   **81** |
|   **82** |
|   **83** |
|   **84** |
|   **85** |
|   **86** |
|   **87** |
|   **88** |
|   **89** |
|   **90** |
|   **91** |
|   **92** |
|   **93** |
|   **94** |
|   **95** |
|   **96** |
|   **97** |
|   **98** |
|   **99** |
|   **100** |
| **101** |
| **102** |
| **103** |
| **104** |
| **105** |
| **106** |
| **107** |
| **108** |
| **109** |
| **110** |
| **111** |
| **112** |
| **113** |
| **114** |
| **115** |
| **116** |
| **117** |
| **118** |
| **119** |
| **120** |
| **121** |
| **122** |
| **123** |
| **124** |
| **125** |
| **126** |
| **127** |
| **128** |
| **129** |
| **130** |
| **131** |
| **132** |
| **133** |
| **134** |
| **135** |
| **136** |
| **137** |
| **138** |
| **139** |
| **140** |
| **141** |
| **142** |
| **143** |
| **144** |
| **145** |
| **146** |
| **147** |
| **148** |
| **149** |
| **150** |
| **151** |
| **152** |
| **153** |
| **154** |
| **155** |
| **156** |
| **157** |
| **158** |
| **159** |
| **160** |
| **161** |
| **162** |
| **163** |
| **164** |
| **165** |
| **166** |
| **167** |
| **168** |
| **169** |
| **170** |
| **171** |
| **172** |
| **173** |
| **174** |
| **175** |
| **176** |
| **177** |
| **178** |
| **179** |
| **180** |
| **181** |
| **182** |
| **183** |
| **184** |
| **185** |
| **186** |
| **187** |
| **188** |
| **189** |
| **190** |
| **191** |
| **192** |
| **193** |
| **194** |
| **195** |
| **196** |
| **197** |
| **198** |
| **199** |
| **200** |
| **201** |
| **202** |
| **203** |
| **204** |
| **205** |
| **206** |
| **207** |
| **208** |
| **209** |
| **210** |
| **211** |
| **212** |
| **213** |
| **214** |
| **215** |
| **216** |
| **217** |
| **218** |
| **219** |
| **220** |
| **221** |
| **222** |
| **223** |
| **224** |
| **225** |
| **226** |
| **227** |
| **228** |
| **229** |
| **230** |
| **231** |
| **232** |
| **233** |
| **234** |
| **235** |
| **236** |
| **237** |
| **238** |
| **239** |
| **240** |
| **241** |
| **242** |
| **243** |
| **244** |
| **245** |
| **246** |
| **247** |
| **248** |
